# A highly active heparinase I from *Bacteroides cellulosilyticus*: Cloning, high level expression, and molecular characterization

**DOI:** 10.1371/journal.pone.0240920

**Published:** 2020-10-20

**Authors:** Li-Wei Gao, Hong-Tao Zhu, Cai-Yun Liu, Zhi-Xiang Lv, Xiao-Man Fan, Ye-Wang Zhang

**Affiliations:** 1 The People’s Hospital of Danyang, Affiliated Danyang Hospital of Nantong University, Danyang, Jiangsu Province, China; 2 School of Pharmacy, Jiangsu University, Zhenjiang, People’s Republic of China; Xinyang Normal University, CHINA

## Abstract

As one of the most extensively studied glycosaminoglycan lyases, heparinase I has been used in producing low or ultra-low molecular weight heparin. Its’ important applications are to neutralize the heparin in human blood and analyze heparin structure in the clinic. However, the low productivity and activity of the enzyme have greatly hindered its applications. In this study, a novel Hep-I from *Bacteroides cellulosilyticus* (BcHep-I) was successfully cloned and heterologously expressed in *E*. *coli* BL21 (DE3) as a soluble protein. The molecular mass and isoelectric point (pI) of the enzyme are 44.42 kDa and 9.02, respectively. And the characterization of BcHep-I after purified with Ni-NTA affinity chromatography suggested that it is a mesophilic enzyme. BcHep-I can be activated by 1 mM Ca^2+^, Mg^2+^, and Mn^2+^, while severely inhibited by Zn^2+^, Co^2+^, and EDTA. The specific activity of the enzyme was 738.3 U·mg^-1^ which is the highest activity ever reported. The *K*_m_ and *V*_max_ were calculated as 0.17 mg·mL^-1^ and 740.58 U·mg^-1^, respectively. Besides, the half-life of 300 min at 30°C showed BcHep-I has practical applications. Homology modeling and substrate docking revealed that Gln15, Lys74, Arg76, Lys104, Arg149, Gln208, Tyr336, Tyr342, and Lys338 were mainly involved in the substrate binding of Hep-I, and 11 hydrogen bonds were formed between heparin and the enzyme. These results indicated that BcHep-I with high activity has great potential applications in the industrial production of heparin, especially in the clinic to neutralize heparin.

## Introduction

Heparinase I (Hep-I) was first isolated from *Pedobacter heparinus* (formerly known as *Flavobacterium heparinum*) [1]. It’s capable of cleaving heparin and heparan sulfate at the 1–4 linkages by β-elimination [2]. And this specific site recognition makes it a useful tool in various fields. It not only can be used for manufacturing low molecular weight or ultra-low molecular weight heparin in industry [3,4], but also can be applied in analyzing heparin structure and contamination [5], eliminating the heparin in human blood [6]. Owing to these important applications in the clinic and industry, Hep-I has been receiving great attention incessantly. Up to now, a variety of Hep-Is in different sources have been identified and characterized from *F*. *heparinum* [7,8], *Bacteroides thetaiotaomicron* [9], *Bacteroides stercoris* [10], and *Sphingobacterium* [11]. However, these native Hep-Is have low expression in the original organisms, and the complexity of separation and purification makes it difficult to obtain enough enzymes for pilot or industrial-scale production.

To improve the expression of Hep-I, it is efficient to express the enzyme heterologously in engineering bacteria. *Escherichia coli* was usually chosen as the host which is one of the most successful and economical systems of expression. The Hep-Is from *F*. *heparinum* [12], *B*. *thetaiotaomicron* [13], and *Bacteroides eggerthii* [14] have been successfully expressed in *E*. *coli*. However, the recombinant enzymes still suffer the problems of an inclusion body and low activity recovery that lead to a low yield [15,16]. Thus, the strategy of forming a fused protein was employed to improve the soluble expression. The fusion tags including cellulose-binding domain (CBD), glutathione-S-transferase (GST), and maltose-binding protein (MBP) have been used for heterologous expression of Hep-I. Nevertheless, the recombinant CBD-Hep-I was still expressed mainly in inactive inclusion bodies [17], while GST-Hep-I had to be expressed at a low temperature to achieve a higher solubility [13]. Although about 90% of the enzyme could be expressed as the soluble form when it was fused as MBP-Hep-I in *E*. *coli* at 15°C, its *K*_m_ was 4.8-fold higher than that of the native enzyme, implying that the affinity of the fused Hep-I towards heparin was strongly hindered the MBP tag [18].

The low activity of all the reported Hep-Is is another bottleneck for the enzyme production [19]. The specific activity of the Hep-I from *F*. *heparinum* was 90.33 U·mg^-1^, and there was only a 54% improvement obtained after the directed evolution [20]. The specific activity of the wild-type Hep-Is from *B*. *stercoris and Sphingobacterium* were only 8.8 and 17.6 U·mg^-1^, respectively [10,11]. Even though the recombinant Hep-I from *B*. *thetaiotaomicron* was 164 U·mg^-1^ which could not meet the requirement of the applications [13]. So, it’s necessary to screen new Hep-I with good stability and high enzyme activity.

In the present work, a novel heparinase I from *Bacteroides cellulosilyticus* was cloned and expressed in *E*. *coli* BL21 (DE3) as the soluble form. The recombinant Hep-I was fused with a hexahistidine-tag (6×His) for efficient purification. And the characterization of the enzyme showed that it has a quite high specific activity for practical applications. The structural analysis of the enzyme with molecular docking was performed to explore the possible mechanism.

## Materials and methods

### Chemicals

Oligonucleotide primers, DNA polymerase for PCR, and T4 DNA ligase were obtained from Beyotime Biotech Co. Ltd (Haimen, China). The PET-28a expression plasmid, DNA extraction kit, and *E*. *coli* DH5α and BL21 (DE3) were purchased from Sangon Bioengineering Co. Ltd (Shanghai, China). Restriction endonucleases and nickel-nitrilotriacetic acid (Ni-NTA) superflow column were provided by Transgen Biotech Co. Ltd (Beijing, China). DNA ladder and protein molecular weight marker were obtained from Detai Biologics Co. Ltd (Nanjing, China). Kanamycin sulfate, sodium heparin, and isopropyl-b-D-thiogalactopyranoside (IPTG) were ordered from Aladdin Co. Ltd (Shanghai, China). All other reagents used in the experiments were analytical grade and supplied by Sigma-Aldrich Co. Ltd (St. Louis, MO).

### Strains and the culture conditions

*B*. *cellulosiliticus* was obtained from Guangdong Microbiology Culture Center (Guangzhou, China) and cultured at 37°C in brain heart infusion (BHI) medium. *E*. *coli* strains DH5α and BL21 (DE3) were cultivated in Luria–Bertani (LB) broth containing 50 μg·mL^-1^ kanamycin sulfate at 37°C.

### Enzyme assay

According to the previous report [14], the enzyme assay was performed by an ultraviolet spectrophotometer at 232 nm. The enzymatic reaction was carried out at 30°C in pH 7.5 Tris-HCl buffers (containing 200 mM NaCl, 10 mM CaCl_2_, and 20 mM Tris-HCl). And the reaction mixture consisted of 895 μL Tris-HCl buffer, 100 μL of 25 mg·mL^-1^ sodium heparin and 5 μL of enzyme solution. Then the activity of enzyme was calculated through monitoring the UV absorbance at 232 nm. And the molar extinction coefficient of the unsaturated uronic acid is 3800 L (mol·cm)^-1^. One international unit was defined as the amount of protein that can form 1 μmol unsaturated uronic acid per minute at 30°C.

### Cloning of the Hep-I from *B*. *cellulosilyticus*

*B*. *cellulosilyticus* genome DNA was extracted using the DNA extraction kit, and it was used as the template for amplification of BcHep-I gene by polymerization chain reaction (PCR) with the primer oligonucleotides listed below: 5’-CGGGATCCGCTGACCGATAAAACCGACC-3’ and 5’-CCTTCGAATCCAGTCAATATCCACCCGG-3’ (*Bam* HI and *Hind* III restriction sites are underlined, respectively). After amplification, the PCR product was digested with the *Bam* HI and *Hind* III endonucleases. Then it was ligated with pET-28a which was digested with same enzymes and recovered from the agarose gel. The recombinant plasmid of pET-28a-Hep-I was followed to be transformed into *E*. *coli* DH5α, and then grown in LB plates containing kanamycin sulfate (50 μg·mL^-1^) and cultured at 37°C. The DNA sequencing and double enzymes digestion were utilized to confirm the positive transformants.

### Expression and purification of BcHep-I

The plasmid possessing an accurate insertion of the Hep-I gene was then transformed into *E*. *coli* BL21 (DE3) host cells to express the recombinant Hep-I. An overnight culture of *E*. *coli* BL21 (DE3) containing pET28(a)-BcHep-I plasmids was inoculated into LB medium containing kanamycin sulfate (50 μg·mL^-1^) and cultivated at 37°C until the optical density at 600 nm (OD_600_) reached 0.6–0.8. Then IPTG was added to a final concentration of 0.2 mM. The induction temperature and time were varied to investigate the expression of the recombinant enzyme by evaluation of the enzyme activity and the soluble protein with sodium dodecyl sulfate-polyacrylamide gel electrophoresis (SDS-PAGE).

The recombinant Hep-I obtained from the cultivated cells was purified by Ni-NTA affinity chromatography. In detail, 100 mL of the culture broth after the IPTG induction was harvested by centrifugation at 5000×g for 5 min, and it was suspended in 5 mL lysis buffer (20 mM Tris-HCl contains 200 mM NaCl, 10 mM CaCl_2_, and 20 mM imidazole, pH 7.5) after washed twice with the same buffer. Cells were disrupted by ultrasonic treatment in an ice bath. The crude enzyme in the supernatant was obtained by centrifugation at 4°C of 5000×g for 20 min to remove cell debris. The supernatant with soluble recombinant Hep-I was loaded to 2 mL of Ni-NTA pre-equilibrated with 10 mL lysis buffer. The column was then washed with 25 mL of the lysis buffer followed by eluted with 2 mL elution buffer (20 mM Tris-HCl contains 200 mM NaCl, 10 mM CaCl_2_, 250 mM imidazole, pH 7.5). The purified Hep-I was analyzed with 12% SDS-PAGE and visualized by staining with Coomassie brilliant blue R250. And the protein concentration was determined with the Bradford method [21].

### Biochemical characterization of BcHep-I

For the characterization of the recombinant BcHep-I, several factors influencing enzyme activity were investigated using sodium heparin as the substrate. The effect of pH on the activity of the recombinant BcHep-I was examined in 20 mM sodium citrate (pH 4.6~6.6), Bis-Tris (pH 6.6~7.1), and Tris-HCl (pH 7.1~9.0) buffers [22]. For the effect of temperature, the enzymatic activities of BcHep-I at a temperature range of 20~60°C were investigated. And the effects of metal ions and ethylene diamine tetraacetic acid (EDTA) on the activity of BcHep-I were studied in Tris-HCl buffers containing 1 mM CaCl_2_, MnCl_2_, CoCl_2_, MgCl_2_, ZnCl_2_, NaCl, LiCl, and EDTA, respectively. The reaction buffer without metal ions was set as the control.

Kinetic parameters (*K*_m_ and *V*_max_) of the recombinant Hep-I for sodium heparin were determined using a series of substrate solutions (0.01~20 mg·mL^-1^) under the conditions of 30°C and pH 7.5. They were calculated with nonlinear regression of the Michaelis–Menten equation. All the experiments were repeated 3 times.

### The thermostability and storage stability of BcHep-I

The thermostability of BcHep-I was examined at 30 and 37°C, respectively. The purified enzyme was incubated at temperatures of 30 and 37°C for different intervals and then immediately taken away at the scheduled time and cooled at 4°C for one minute before the assay of the residual activity. The storage stability of BcHep-I at 4°C was studied as well. The purified enzyme was dispensed into several 1.5 mL Eppendorf tubes, then directly kept at 4°C, and the relative activity of BcHep-I was calculated.

### Homology modeling and substrate docking

Protein sequence analysis showed that BcHep-I shared 65.8% identity with the Hep-I from *B*. *thetaiotaomicron*, and multiple sequence alignment of several heparinases I was performed with ClustalW [23] and shown with Jalview. A three-dimensional model structure of BcHep-I was built using the automated protein structure homology modeling server Swiss-Model [24] according to the known crystal structure of the BtHep-I (3ikw). The reliability of the model was verified by PROCHECK [25]. Adding polar hydrogens, assigning Gasteiger charges to all atoms of the heparin and docking of the substrate into the active site of BcHep-I were all performed with AutoDock4 [26]. Structural representations were generated using PyMOL [27].

## Results and discussion

### Sequence analysis of the Hep-I from *B*. *cellulosilyticus*

**Fig 1:**
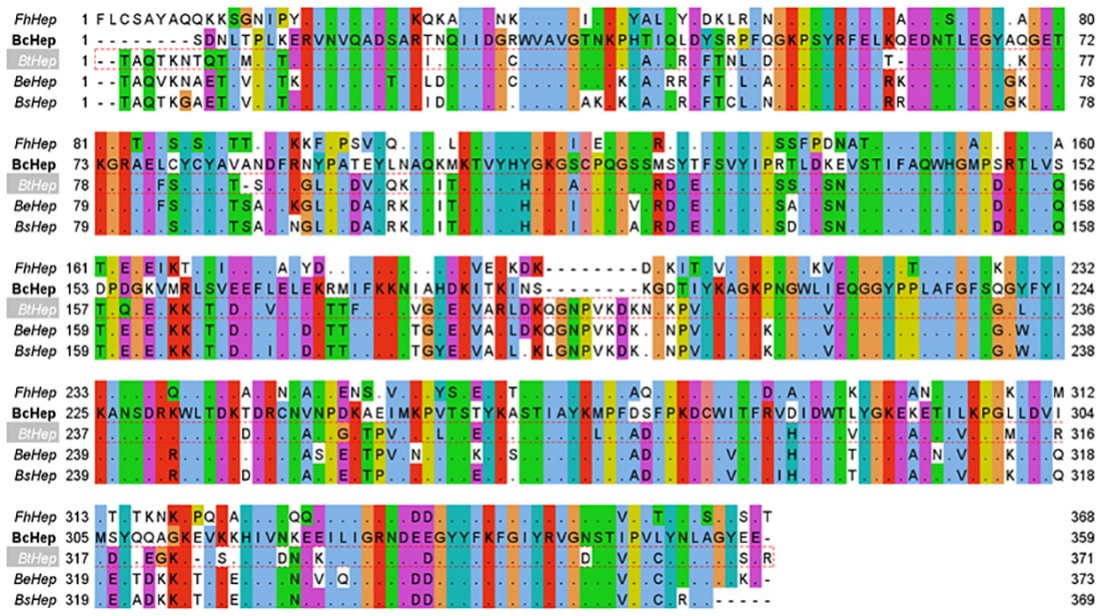
Multiple sequence alignment of heparinases I from *F*. *heparinum* (FhHep-I), *B*. *cellulosilyticus* (BcHep-I), *B*. *thetaiotaomicron* (BtHep-I: PDB ID: 3ikw), *B*. *eggerthii*, and *B*. *stercoris* (BsHep-I). The residues of BcHep-I and the nonconserved residues of other Hep-Is were shown and it was generated with JalView (2.11.1.2).

The full-length gene encoding Hep-I of *B*. *cellulosilyticus* was 1169 bp and encoded 389 amino acids. And the protein sequence exhibits 63.1%, 65.8%, 63.3%, and 62.8% identities with the Hep-Is from *F*. *heparinum*, *B*. *thetaiotaomicron*, *B*. *stercoris*, and *B*. *eggerthii*, respectively. Multiple sequence alignment of Hep-I from various species was presented in Fig 1. To our knowledge, Cys135 and His203 are the vital nucleophile amino acids of FhHep-I in the endocytic cleavage of heparin [28], and Lys199 is one of two calcium-binding sites which are necessary for the maintenance of enzymatic activity of FhHep-I [29]. It shows that all of them are highly conserved in BcHep-I, and may have a certain effect on the structure and catalytic activity. The molecular mass of encoded protein is 44.42 kDa as calculated by ExPASy, with an isoelectric point (pI) of 9.02.

### Identification, expression, and purification of BcHep-I

**Fig 2:**
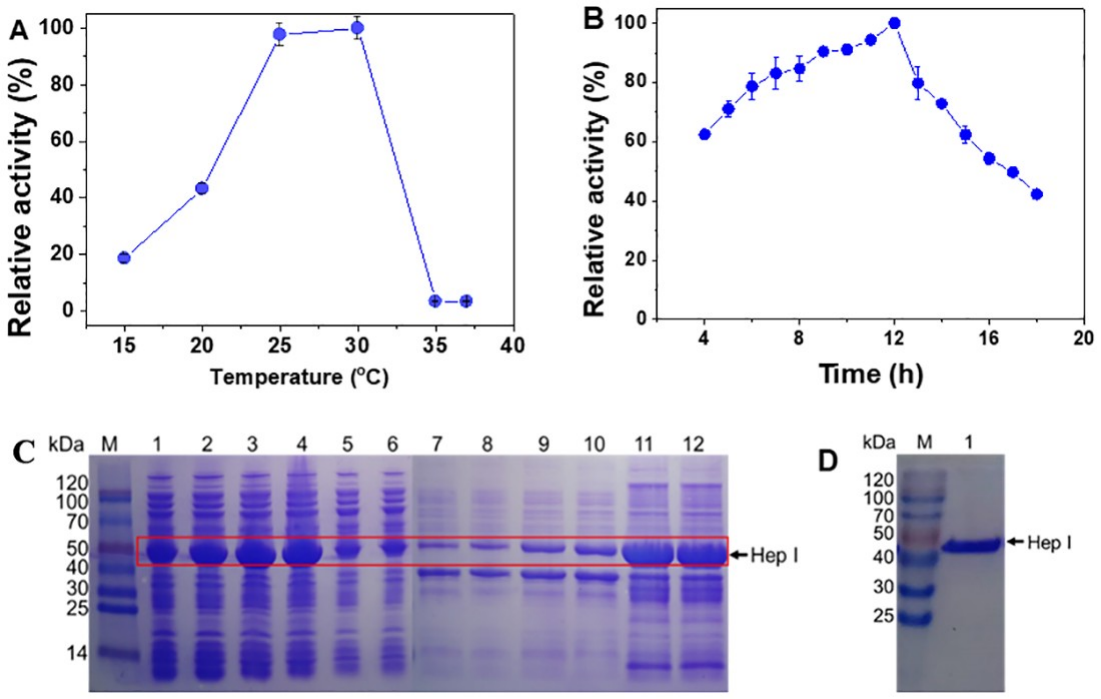
Effect of induction temperature on the activity (A) and the expression (B) (SDS-PAGE image of the BcHep-I at different temperatures. M: protein molecular weight marker, lane 1~6: supernatant after the sonication of the IPTG induced *E*. *coli* at 15, 20, 25, 30, 35, and 37°C; lane 7~12: precipitate after the sonicated of the IPTG induced *E*. *coli* at 15, 20, 25, 30, 35, and 37°C; BcHep-I bands were marked with solid red wireframe) of BcHep-I. Effect of the induction time on the activity of BcHep-I (C). SDS-PAGE of the purified Hep-I (D) (M: protein molecular weight marker, lane 1: the purified BcHep-I).

The expression plasmid was subjected to sequence and it proved that construction of the pET-28a-Hep-I was successful. Then the expression of BcHep-I in *E*. *Coli* BL21 (DE3) was investigated at different induction temperatures and time shown in Fig 2. Here, the induction temperature was varied from 15 to 37°C under the conditions of 0.2 mM IPTG and 7 h induction. As shown in Fig 2A, it’s obvious that the activity of Hep-I increased from 18.7% to 100% with the increase of the induction temperature from 15 to 30°C, and then it decreased rapidly to 3.5% when the temperature continuously increased to 37°C. The highest activity was obtained at 30°C and set as 100%. Besides, more than 90% of the enzyme was found to be present as the soluble form at the induction temperatures lower than 30°C (Fig 2B). However, the expression form of BcHep-I enzyme was mainly an inclusion body without enzyme activity at 35 and 37°C. This is because the recombinant protein was expressed fast at high temperatures and cannot fold correctly leading to form the inclusion body [30,31]. Therefore, a temperature ranged from 25 to 30°C was suitable for the expression of BcHep-I as the soluble protein.

The effect of induction time on the activity of the enzyme was investigated in Fig 2C. When the induction time was increased to 12 h, the activity of the recombinant enzyme reached the highest value because of the accumulation of the recombinant enzyme. Then with the prolonging of induction, the enzyme activity started to decrease. This might be that the expression of the recombinant enzyme was decreased with the consumption of the nutrients in the medium [32]. Besides, the level of the recombinant proteins expression is system-specific and may be influenced by other factors, like the host strain and cultivation conditions, especially the biochemical properties of the recombinant protein.

The affinity purification of the soluble recombinant Hep-I was shown in Fig 2D. The recombinant Hep-I could be separated well by one-step affinity purification using Ni-NTA chromatography. After purification, an apparent single Hep-I band was found in the SDS-PAGE image, in accordance with the calculated molecular weight of 44.42 kDa.

### Biochemical characterization of BcHep-I

**Fig 3:**
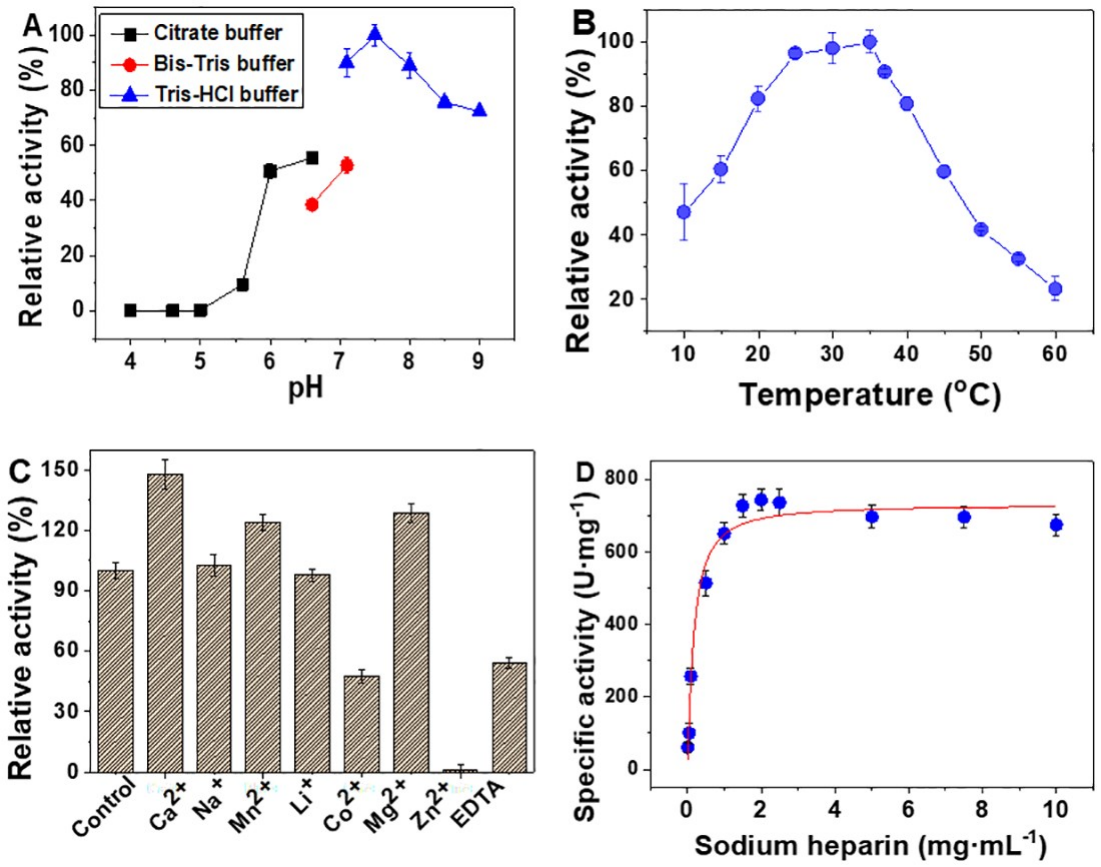
Effects of pH (A), temperature (B), metal ions and EDTA (C), and (D) substrate concentration (D) on the activity of the Hep-I. The enzyme activity was measured according to the enzyme assay and every experiment was repeated three times.

The effect of pH on the activity of BcHep-I for sodium heparin was described in Fig 3A. The highest activity of BcHep-I at pH 7.5 was set as 100%. It can be seen that BcHep-I almost had no activity between pH 4 to 5, and with the pH increased from 5.6 to 7.5, the relative activity of the enzyme increased sharply from 7.7% to 100%. Then the activity of enzyme decreased when the pH increased continuously. Another notable phenomenon is that the activity of BcHep-I was influenced by different buffers. Activities in citrate and Tris-HCl buffer were 17% and 37.21% higher than that in Bis-Tris buffer, respectively. Overall, the enzyme displayed more than 89% relative activity in the pH range 7.1~8.0 in Tris buffer. It means that BcHep-I has a broader working pH than other Hep-Is including BtHep-I, FhHep-I, and BsHep-I with an optimal pH of 7.0 [7,10,13].

The effect of temperature on the enzyme activity of BcHep-I was shown in Fig 3B. The activity increased with the temperature from 10 to 35°C and the highest specific activity of 738.3 U·mg^-1^ was obtained at 35°C. Then the activity of BcHep-I declined rapidly when the temperature raised from 37 to 60°C and there is no detectable activity at 60°C. It was due to the stability of the enzyme decreased under higher temperatures and its steric structure was destroyed which resulted in the decrease of enzyme activity and even inactivation [33].

The activity of BcHep-I was significantly influenced by divalent cations and EDTA [34]. In Fig 3C, the activity of BcHep-I was enhanced by 1 mM Mn^2+^ (1.23-fold), Mg^2+^ (1.28-fold), Ca^2+^ (1.48-fold). However, 1 mM EDTA (0.47-fold) or Co^2+^ (0.54-fold) inhibited BcHep-I, and Zn^2+^ could inactivate the enzyme, whereas Na^+^ and Li^+^ had no obvious influence on the activity of BcHep-I. According to the previous study [35], there are two identified sites (CB-1 and CB-2) for the binding of heparinase I and calcium, which play an important role in the catalysis reaction of Hep-I. Because it has been reported that calcium ions play an important role in facilitating bridging between the enzyme and substrate in the enzyme-substrate complex, and this interaction is critical for the proper enzymatic function of Hep-I.

The Michaelis–Menten constant was determined with different concentrations of sodium heparin (Fig 3D). With the concentration increased to 2 mg·mL^-1^, the activity of BcHep-I rapidly increased to the maximum. The *K*_m_ and *V*_max_ values of BcHep-I were 0.17 mg·mL^-1^ and 740.58 U·mg^-1^ through nonlinear regression calculation, respectively. It is worth noting that the enzyme has the highest *V*_*max*_ ever reported, which is 84-fold of BsHep-I [10], 17.3-fold of BtHep-I [13], 8.2-fold FhHep-I [7], and 4.5-fold of SpHep-I (Table 1). In our previous work, a Hep-I from *B*. *eggerthii* was expressed with a remarkable *V*_*max*_ of 647.93 U·mg^-1^. Compared with BeHep-I, there was around 15% improvement of the V_max_. However, the *K*_*m*_ of BcHep-I was only 0.047-fold of BeHep-I, which means BcHep-I has much higher catalytic efficiency. In brief, the lower *K*_*m*_ and higher *V*_*max*_ indicated that the BcHep-I has a better affinity with substrate and higher enzymatic reaction rate leading to a more efficient conversion of heparin. This advantage also could be proved from the fact that the specific activity of BcHep-I was 54% higher than that of BeHep-I. It will benefit the application in the clinic because high activity Hep-I will significantly reduce the dose of the enzyme during the neutralization of heparin in human blood, and might lower the risk of immunological rejection.

**Table 1:** Comparison of the activity, half-life and kinetic parameters of heparinases I from different organisms.

Organisms	Specific activity (U·mg^-1^)	t_1/2_ (min)		Kinetic parameters		References
		30°C	37°C	*K*_m_ (mg mL^-1^)	*V*_max_(U·mg^-1^)	
*F*. *heparinum*	90.33	10	-	17.8	219.48	[7,8]
*B*. *thetaiotaomicron*	164	-	-	2.3	42.7	[13]
*B*. *stercoris*	16	-	-	13	8.8	[10]
*Sphingobacterium*	17.6	-	-	42	166	[11]
*B*. *eggerthii*	480	350	60	3.6	647.93	[14]
*B*. *cellulosilyticus*	738.8	300	59	0.17	740.58	This work

### The thermal and storage stability of BcHep-I

**Fig 4:**
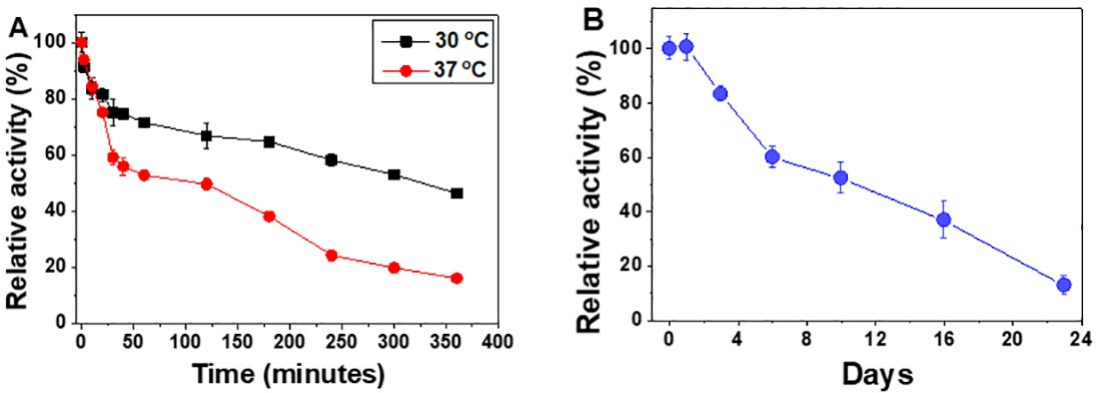
The thermostability of BcHep-I at 30 and 37°C (A) and the storage stability at 4°C (B). The enzyme activity was determined according to the enzyme assay and every experiment was repeated three times.

The stability (Fig 4) is an important property for the applications of Hep-I. The thermal stability of the purified BcHep-I was examined at 30 and 37°C, respectively. As shown in Fig 4A, the relative activity of BcHep-I decreased by 36% in the first 40 minutes at 30°C, and then declined slowly and remained 46% of the initial activity after 360 min incubation. The half-lives of BcHep-I were 300 and 59 min at 30 and 37°C, which were similar to that of BeHep-I (350 and 60 min at 30 and 37°C, respectively) [14]. These thermostability results exhibited that the heparinase I from *B*. *cellulosilyticus* has practical applications in the clinic and industry.

The storage stability at 4°C was also studied (Fig 4B). There were100 and 81% of its initial activity retained after storing at 4°C for 1 and 3 days, and only remained 12% after the storage of 23 days. The half-life of the enzyme is about 10 days which is much better compared with other heparinases I. Therefore, BcHep-I has advantages in storage and transportation for good storage stability.

### Homology modeling and substrate docking

**Fig 5:**
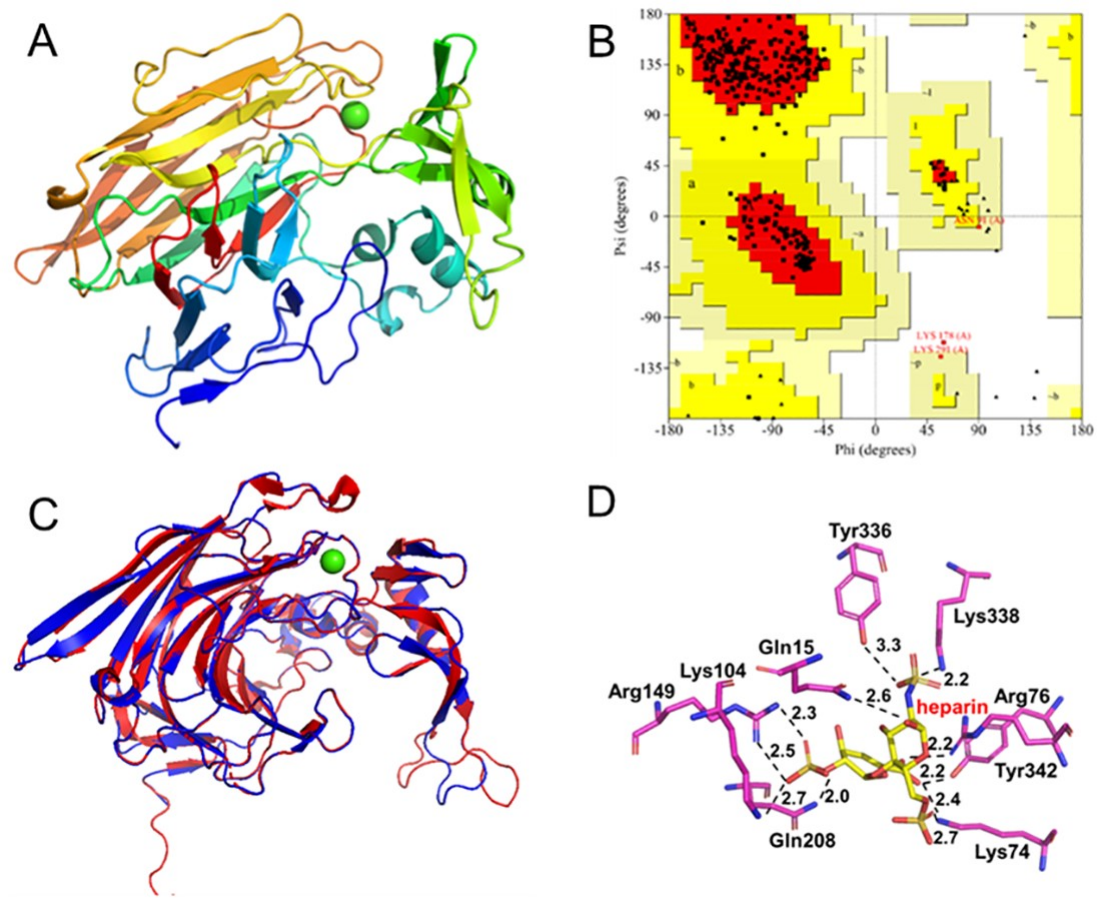
Homology modeling of BcHep-I based on the crystal structure of 3ikw and molecular docking with heparin. (A: the homology model of BcHep-I; B: Ramachandran plot of BcHep-I, C: The superimposition of the template (BtHep-I, 3ikw, red) and BcHep-I (blue), D: molecular docking of BcHep-I with heparin). The images were produced with PyMOL (2.5) and AutoDockTools (1.5.7).

The homology model of the BcHep-I was built using the known crystal structure of BtHep-I by Swiss-Model (PDB ID: 3ikw), shown in Fig 5A. The quality of the model was evaluated by SAVES online server, and the values of 94.203 indicated that it was quite good. The Ramachandran map demonstrated that 92% (288) of residues located in the most favored regions, 7% (22) residues in additional favored regions, 0.6% (2) residues in generally favored regions, and 0.3% (1) residues in disallowed regions (Fig 5B). The result of Verified 3D showed that the mean 3D-1D score of 93.31% residues was greater than 0.2. All results suggested that the structure of the model was fine enough and suitable for further docking.

As described, structural representations were generated using PyMOL. The superimposition of BcHep-I and BtHep-I (Fig 5C) showed that BcHep-I has a similar steric structure with BtHep-I and both enzymes bind calcium ion with Trp, Asn, Asp, and Glu residues. The substrate-binding involved many direct and water-mediated hydrogen bonds between the sulfate groups and the basic side chains within the active site [36,37]. The critical residues in active pockets of the Hep-I were shown in Fig 5D. These residues including Gln15, Lys74, Arg76, Lys104, Arg149, Gln208, Tyr336, Tyr342, and Lys338 connected the heparin by forming hydrogen bonds. Several residues including Gln15, Arg76, Lys104, Gln208, Tyr336, Lys338, and Tyr342 conjugated the heparin with 2.6 Å, 2.2 Å, 2.7 Å, 2.0 Å, 3.3 Å, 2.2 Å, and 2.2 Å of hydrogen bonds, respectively. While Lys74 formed 2.7 Å and 2.4 Å of hydrogen bonds to different atoms of heparin, so as Arg149 formed two hydrogen bonds of 2.7 Å and 2.5 Å. In total, there are 11 hydrogen bonds formed between 9 residues of BcHep-I and heparin. These hydrogen bonds contribute to the stabilization of the enzyme-substrate complex, and the results were consistent with the conformational entropy of BcHep-I (762 J·K^-1^), which is lower than that of the other heparinases I (756 J·K^-1^ of BeHep-I, 768 J·K^-1^ of FhHep-I, 782 J·K^-1^ of BsHep-I and 774 J·K^-1^ of BtHep-I, respectively). These evidences proved the high activity of BcHep-I could attribute to its unique molecular structure. Taking all the results into account, the stability of BcHep-I could be further promoted which may be achieved through protein engineering or immobilization [38–40] in the future. Therefore, the application prospect of heparinase I is broad and promising.

## Conclusions

In summary, a highly active Hep-I from *B*. *cellulosilyticus* was successfully cloned and heterologously expressed in *E*. *coli* BL21 (DE3). The recombinant BcHep-I has a specific activity of 738.3 U·mg^-1^ which is the highest activity ever reported to the best of our knowledge. The half-life of BcHep-I exhibits reasonable thermal and storage stability which would benefit the storage and transportation of the enzyme. Consideration of the excellent *K*_*m*_ and *V*_*max*_ of the recombinant enzyme, BcHep-I has great potential in the clinic as medicine and industrial production of low or ultra-low molecule weight heparin.

## Supporting information

S1 Graphical abstract (TIF)
