# Supplementary figures and images for: A highly active heparinase I from Bacteroides cellulosilyticus: Cloning, high level expression, and molecular characterization
Source: PLoS One. 2020 Oct 20;15(10):e0240920. doi: 10.1371/journal.pone.0240920 (PMC7575093; doi:10.1371/journal.pone.0240920)

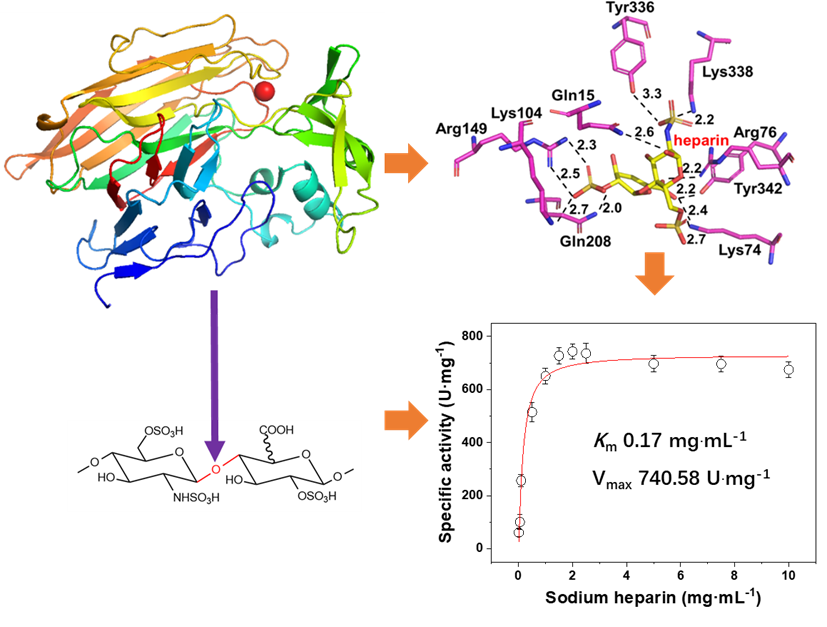

Supplement: S1 Graphical abstract — (TIF) [file pone.0240920.s001.tif]
